# Metal Ion-Catalyzed Low-Temperature Curing of Urushiol-Based Polybenzoxazine

**DOI:** 10.3389/fchem.2022.879605

**Published:** 2022-04-28

**Authors:** Wen Yang, Yaofeng Xie, Jipeng Chen, Chunmei Huang, Yanlian Xu, Yucai Lin

**Affiliations:** ^1^ College of Chemistry and Materials, Fujian Normal University, Fuzhou, China; ^2^ Fujian Engineering Research Center of New Chinese Lacquer Materials, Minjiang University, Fuzhou, China; ^3^ Fujian Key Laboratory of Polymer Materials, Fujian Normal University, Fuzhou, China; ^4^ Fujian Provincial Key Laboratory of Advanced Oriented Chemical Engineering, Fujian Normal University, Fuzhou, China

**Keywords:** polybenzoxazine, urushiol, metal ions catalyst, curing temperature, metal coordination

## Abstract

In this work, urushiol-based polybenzoxazine is cured by the Lewis acid (FeCl_3_, AlCl_3,_ and CuCl_2_) at low temperature instead of high thermal curing temperature. The effect of the Lewis acid on structures and properties of the polymers is revealed. The relating urushiol-based benzoxazine monomer (BZ) was synthesized by natural urushiol, formaldehyde, and *n*-octylamine. The monomer was reacted with the Lewis acid with a molar ratio of 6:1 (N_monomer_: N_Metal_) at 80°C to obtain films that can be cured at room temperature. The chemical structures of benzoxazine monomers were identified by Fourier-transform infrared spectroscopy (FTIR) and ^1^H nuclear magnetic resonance spectroscopy (^1^H-NMR). The interaction between the metal ion and the polymers is revealed by X-ray photoelectron spectroscopy (XPS) and attenuated total reflectance-FTIR (ATR-FTIR). The effect of the Lewis acid on the mechanical properties, wettability, and thermal stability was investigated. The results show that the benzoxazine cured by Cu^2+^ has a better performance than that cured by Al^3+^ and Fe^3+^.

## Introduction

Polybenzoxazine resins, as a new type of phenolic resin (Takeichi et al., 2008), have attracted significant attention due to their unique and excellent properties, such as high mechanical strength, high-temperature performance, low volatile formation, near-zero volumetric change upon curing, low surface energy, low flammability, good electrical resistance, low water absorption, and high char yield. They remain stable to moisture, chemicals, and other corrosive materials. Due to their outstanding and versatile properties, polybenzoxazine resins are widely used in aerospace, automotive, electronic manufacturing, and the preparation of high-performance composites (Ishida and Froimowicz, 2017; Mohamed and Kuo 2020; Samy et al., 2020; Mohamed et al., 2021; Chen et al., 2021a; Mohamed et al., 2022).

Benzoxazine monomers are commonly synthesized by primary amine, phenol, and formaldehyde using the Mannich condensation reaction (Tavernier et al., 2020). One of the most attractive features of benzoxazine resins is their molecular structure design flexibility. Introduction of various functional groups at the amine or phenol fragment can bring new functionalities to materials such as self-healing (Arslan et al., 2018), self-cleaning (Bai et al., 2020), shape memory (Zhang et al., 2018; Hombunma et al., 2020), flame-retardant characteristics (Appavoo et al., 2020; Ding et al., 2022), photo-sensing properties (El-Mahdy et al., 2019), etc. On the other hand, bio-based benzoxazines can be prepared by bio-based phenols and amines instead of petroleum-based ones. Until now, bio-based polybenzoxazine (Salum et al., 2018; Zhan et al., 2019; Lu et al., 2020; Zhan et al., 2020; Cai et al., 2021; Machado et al., 2021; Zhang et al., 2022) resins have been successfully prepared by natural phenols (cardanol (Appavoo et al., 2020), urushiol (Chen et al., 2021b), eugenol (Zhu et al., 2019), resorcinol (Arnebold et al., 2014), guaiacol (Phalak et al., 2017), bisguaiacol F (Periyasamy et al., 2016), phloretic acid (Kirubakaran et al., 2020) (Zhang et al., 2019a), and resveratrol (Zhang et al., 2019b)) and natural amines [(Zhu et al., 2020a), stearyl amine (Zhao et al., 2022), and rosin-based amines (Liu et al., 2017) and (Alhwaige et al., 2019)].

Generally, the benzoxazine monomer can be cured by ring-opening polymerization with thermal treatment. However, the curing temperature of most benzoxazine precursors is high (i.e., over 180°C), which greatly limits the application of polybenzoxazine resins. Much efforts have been devoted to reducing the curing temperature *via* intramolecular interaction [modification of monomer structures by electron-donating or -withdrawing groups, designing of monomer structure to influence the intermolecular packing (rigid groups)] (Xu et al., 2015; Zhang et al., 2019c; Zhu et al., 2020b) or intermolecular interaction cationic initiators including ordinary acids, thiols or elemental sulfur, brønsted acids; catalysts including Lewis acids, amines, latent catalysts, and nanomaterials) (Wang and Ishida 1999; Zhu et al., 2020a; Chen et al., 2021a; Liu et al., 2021; Lochab et al., 2021; Yan et al., 2021). Among these approaches, the introduction of Lewis acid is the most convenient and versatile one. PCl_5_ (Zhang et al., 2021), PCl_3_, POCl_3_, TiCl_4_, and AlCl_3,_ and transition metal salts (Sudo et al., 2010; Ran et al., 2012; Pei et al., 2021; Guorong et al., 2022) like CuCl_2_, AgCl, ZnCl_2_, NiCl_2_ have been introduced into polybenzozine resins (Zhang et al., 2019a; Zhang et al., 2020). They can reduce the curing temperature to some extent. However, the interaction between the metal ions and the polymers and the effect of the metal ion on the properties of the polymers are still not clear. In this work, urushiol-based polybenzoxazine was cured with the Lewis acid (FeCl_3_, AlCl_3_, and CuCl_2_) at a low temperature (∼80°C). The interaction between the metal ions and the polymers was revealed and the effects of the metal ions on the properties of the polymer resin were investigated.

## Experimental Section

### Materials

Chinese lacquer was purchased from Xi’an Institute of Lacquer, China. Urushiol was extracted from Chinese lacquer using ethanol according to the literature. Ferric chloride, copper chloride and aluminum chloride, *n*-octylamine, formaldehyde (37 wt% in H_2_O), 1,4-dioxane, chloroform, dichloromethane, xylene, methanol, and anhydrous sodium sulfate were obtained from Sinopharm Chemical Reagent Co. All chemicals were used as received without further purification.

### Characterization

^1^H Proton nuclear magnetic resonance (^1^H-NMR) spectra were recorded on a Bruker AV400 NMR spectrometer using CDCl_3_ as the solvent and tetramethylsilane (TMS) as the internal standard at a proton frequency of 400 MHz. Fourier transforms infrared (FTIR) spectra were recorded on a United States. Nicolet Magna 5700 spectrometer at room temperature (20°C). The spectra were collected at 32 scans with a spectral resolution of 4 cm^−1^. Reflex spectra were obtained from the method of ATR. Differential scanning calorimetry (DSC) was performed on a METTLER DSC3 instrument under a nitrogen atmosphere at a heating rate of 10°C min^−1^ in the range of 30–250°C. Thermogravimetric analysis (TGA) was performed on a METTLER TGA/SD-TA851 instrument under a nitrogen atmosphere at a heating rate of 10°C min^−1^ in the range of 30–600°C. X-ray photoelectron spectroscopy (XPS) recorded on a VG MultiLab 2000 spectrometer to Mg K_α_ (1,253.6 eV) as X-ray radiation and by being able to 20 eV, using the surface contamination carbon C_1s_ binding energy (284.8 eV) as the internal standard calibration of other elements binding energy. The peak area was obtained by integrating the spectral characteristics of the elements and the sensitivity factor to calculate the composition of each surface element in the catalyst.

### Synthesis of Urushiol-Based Benzoxazine Monomer

To a 100 ml three-necked round bottom flask equipped with a thermometer, a reflux condenser, and a dropping funnel, 10 ml of 1,4-dioxane, formaldehyde (37 wt%) (0.05 mol, 4.05 g), *n*-octylamine (0.025 mol, 3.23 g), and 10 ml of dioxane were added and stirred at below 4°C for 40 min. After adding a solution of urushiol (0.025 mol, 7.85–8.00 g) in 10 ml 1,4-dioxane dropwise within 20 min, the solution was gradually heated to 90°C, and the brown mixture was refluxed for 5 h. The solvent was removed by distillation under reduced pressure, and the residual was dissolved in 100 ml of dichloromethane. The solution was washed many times with distilled water and dried with anhydrous magnesium sulfate for 12 h. The solvent was removed by rotary evaporation, and the brown product was dried under vacuum at room temperature for 24 h. Further purification was conducted by column chromatography and a light red liquid was obtained (yield 96%).

### Synthesis of Urushiol-Based Polybenzoxazine by Metal Ionic Catalyst

A certain amount of urushiol-based benzoxazine monomer (BZ, 0.01 mol) and anhydrous xylene (10 ml) were added to a 100 ml three-necked round bottom flask equipped with a thermometer and a reflux condenser. The solution was stirred under nitrogen for 20 min. Metal chloride (MCl_3_ = FeCl_3_, AlCl_3_ and CuCl_2_) with different molar ratio to BZ (see Table 1) in anhydrous methanol (5 ml) was added dropwise. After vigorous stirring at 80°C for 3 h, PBZ-M (PBZ-Fe, PBZ-Al, and PBZ-Cu) were obtained. PBZ-M (2 ml) was cast onto the clean glass slide or the iron plate and then cured at ambient temperature for 3 h. PBZ cured without metal ions at 120°C (2 h), 140°C (2 h), 160°C (2 h), and 180°C (2 h) was also prepared for comparison. PBA and PBZ-M membranes with a thickness of ∼60 μm were obtained.

**TABLE 1:** Optimization of the reaction ratio of MCl_x_ and BZ.

M	Monomer	Sample	N_monomer_:N_M_	Product	Surface Drying Time
0	BZ	PBZ	—	Reddish-brown liquid	—
FeCl_3_	BZ	PBZ-Fe	3:2	Black power	—
			3:1	Black solid	—
			6:1	Membrane	30 min
			12:1	Membrane	>48 h
AlCl_3_	BZ	PBZ-Al	3:2	Black solid	—
			3:1	Membrane	20 min
			6:1	Membrane	30 min
			12:1	Membrane	>48 h
CuCl_2_	BZ	PBZ-Cu	3:3	Black power	—
			2:1	Black solid	—
			4:1	Membrane	30 min
			8:1	Membrane	>48 h

### Gel Fraction

To calculate the gel fraction of the polymers, the polymers were immersed in toluene for 2 h and then dried in the oven. The gel fraction was calculated as:

Gel fraction = m_t_/m_0_×100%,

where m_0_ is the initial mass of the polymeric film and m_t_ is the mass after immersion.

## Results and Discussion

### Synthesis of Urushiol-Based Polybenzoxazine

**FIGURE 1:**
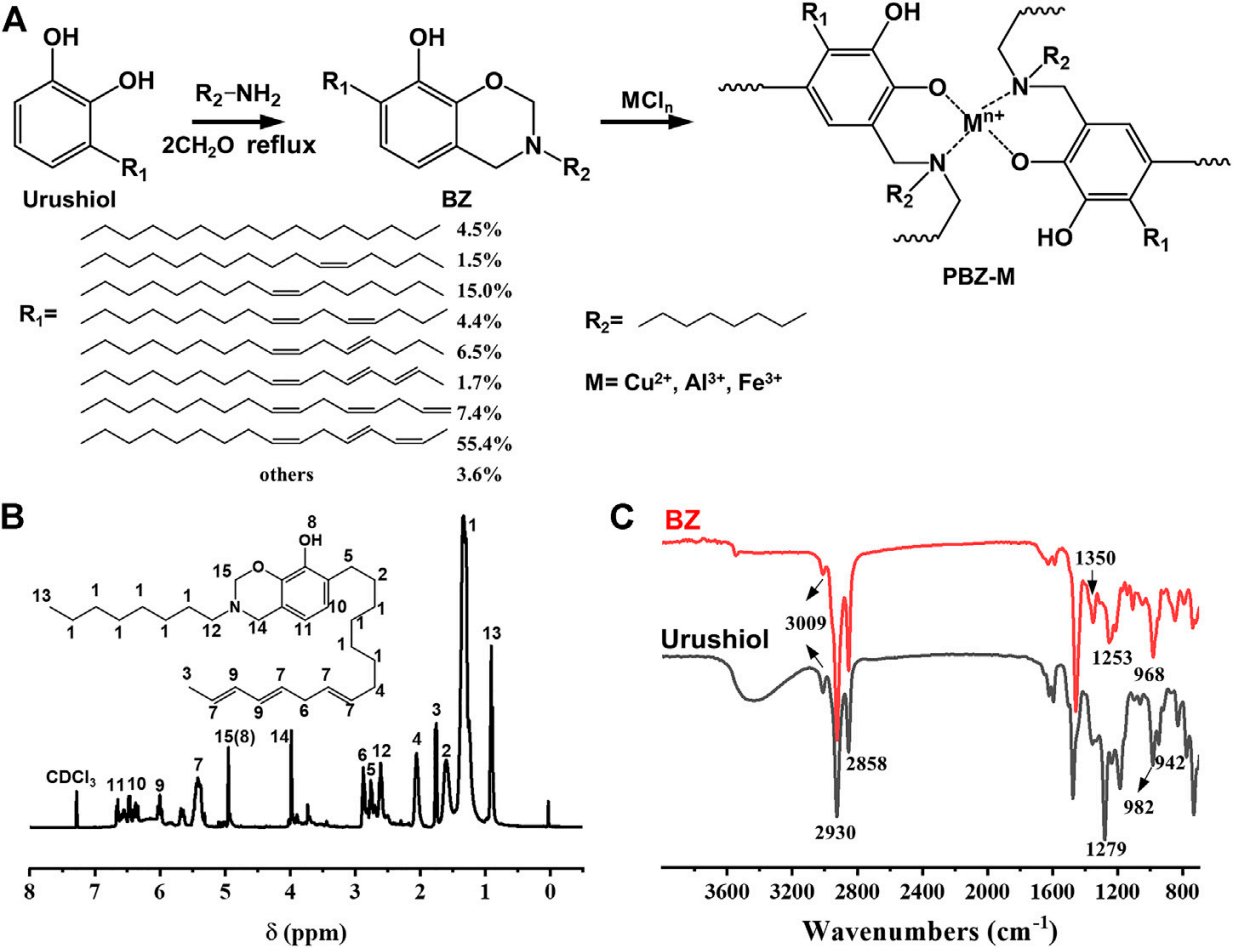
**(A)** Synthesis of BZ and PBZ-M. **(B)** ^1^H-NMR spectrum of BZ. **(C)** FTIR spectrum of urushiol and BZ.

The synthesis of urushiol-based benzoxazine precursor is shown in Figure 1A. The novel benzoxazine monomer was prepared *via* the Mannich reaction of urushiol, *n*-octylamine, and formaldehyde (37 wt% in H_2_O). The reaction time was determined by thin-layer chromatography (TLC). The benzoxazine monomer was reacted with the Lewis acid (FeCl_3_, AlCl_3_, and CuCl_2_) to obtain PBZ-Fe, PBZ-Al, and PBZ-Cu, respectively. The benzoxazine monomer was also thermally cured (PBZ) for comparison. It is worth noting that the polybenzoxazine/MCl_3_ can be cured at ambient temperature. For better performance, the molar ratio of the reactants was optimized. The results are shown in Table 1. The amount of MCl_3_ has a direct impact on the performance of the film. When the amount of MCl_3_ is large, the product is precipitate which could not be coated. When the amount of MCl_3_ is small, the product is viscous liquid while the surface drying time is too long. The suitable molar ratio for film coating is preferable from Table 1 when the ratio of BZ and MCl_3_ including n (BZ): n (FeCl_3_) = 6:1, n (BZ): n (AlCl_3_) = 3:1, n (BZ): n (AlCl_3_) = 6:1, n (BZ): n (CuCl_2_) = 4:1. The molar ratio of BZ and MCl_3_ was kept at 6:1 in the following experiments.

### Characterization of the Benzoxazine Monomer

The structure of the benzoxazine monomer was confirmed by ^1^H-NMR and FTIR. Supplementary Figure S1 shows the ^1^H-NMR and ^13^C-NMR spectrum of urushiol. Figure 1B and Supplementary Figure S2 show the ^1^H-NMR and ^13^C-NMR spectrum of BZ. The peaks in the range 5.40–6.60 ppm are assigned to the protons in–CH = CH–CH = CH–. The peaks from 1.29 to 3.00 ppm are assigned to protons of the saturated bond in the alkyl side group of the urushiol. Two characteristic resonances centered at 4.01 and 4.99 ppm are attributed to the protons in Ar–CH_2_–N and O–CH_2_–N, respectively, which is clear evidence for the formation of benzoxazine. It is worth noting that the proton resonance peak of the oxazine ring (O–CH_2_–N) is overlapped with the phenol hydroxyl. Figure 1C shows the FTIR spectra (Xu et al., 2012) of urushiol and BZ at room temperature. The bands at 947 and 983 cm^−1^ in the spectra of BZ and urushiol are assigned to the bending vibration of the conjugated double bond (-CH = CH-CH = CH-), indicating that the side chain of the urushiol was not changed during the reaction. The bands at 1,621 cm^−1^, 1,595 cm^−1^ in the spectra of urushiol and BZ are assigned to the skeleton vibration absorption peak of the benzene ring (-C=C-). The band at 3,009 cm^−1^ is assigned to the stretching vibration of the isolated double bond (-CH = CH-CH_2_-CH = CH-). The broad band at 3,200–3,600 cm^−1^ was due to the (–OH) vibration. The band at 1,140 cm^−1^ was the asymmetric stretching vibration absorption peak of (C-N-C). In comparison with urushiol, the absorption peaks at 983–947 cm^−1^ shift into one spike, and the absorption is enhanced. BZ exhibited a band at 965 cm^−1^ corresponding to the out-of-plane (-C-H) vibration of the benzene ring to where the oxazine ring is attached and a band at 1,253 cm^−1^ due to the asymmetric stretching of (-C-O-C-) of benzoxazine. In the fingerprint area of the spectra, the band 965 cm^−1^ is the characteristic benzene ring mode of benzoxazine. That broad band at 3,200–3,600 cm^−1^ assigned to the (–OH) vibration is clearly weakened. Both the ^1^H-NMR and FTIR spectra indicate the successful synthesis of the urushiol-based benzoxazine monomer.

### Thermal Curing Behavior of BZ/MCl_x_

**FIGURE 2:**
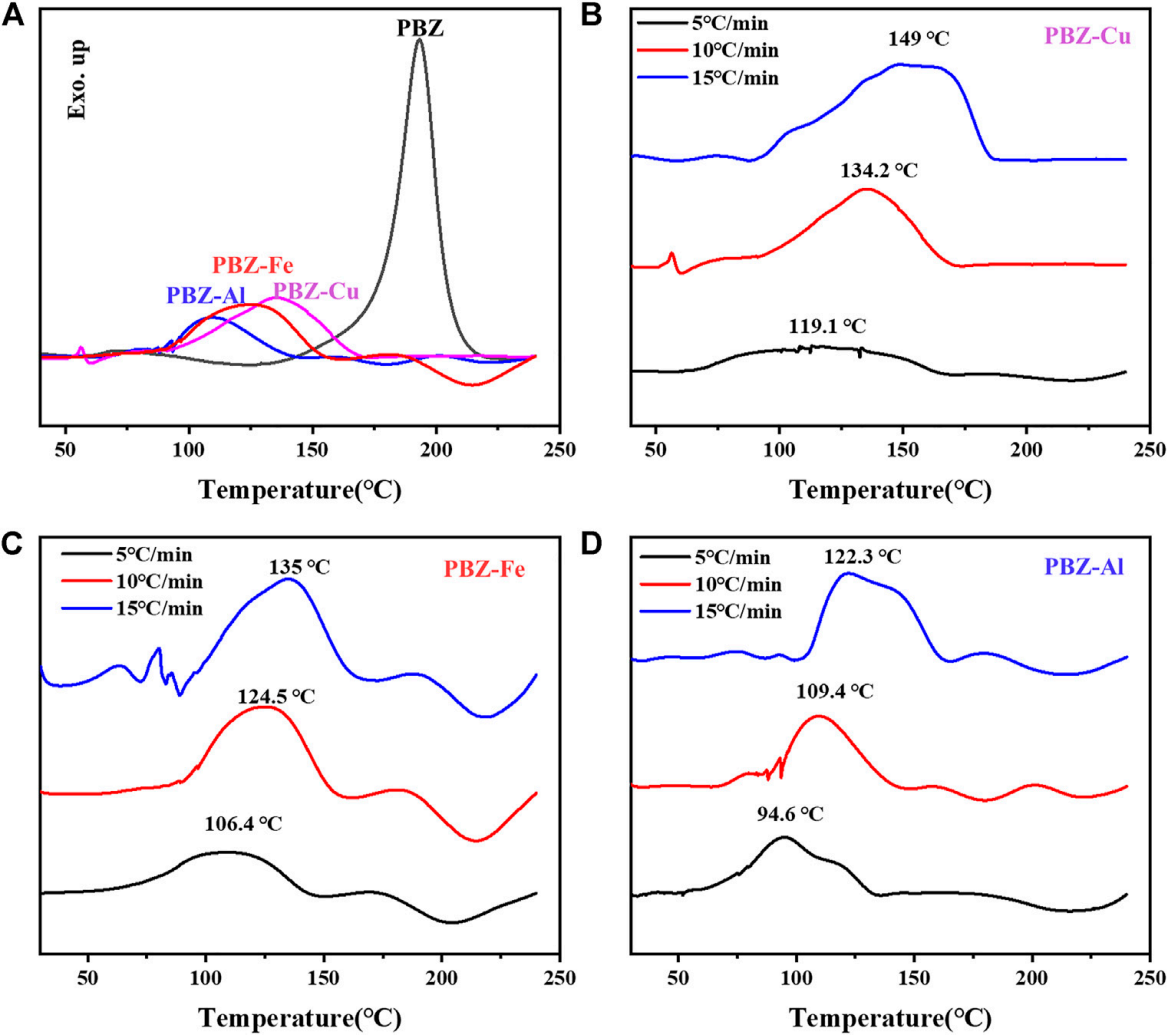
**(A)** Thermal curing behavior of BZ and BZ/MCl_x_. **(B–D)** the curing kinetics of BZ/MCl_x_ at different rates (β = 5, 10, 15°C min^−1^).

The curing behavior of BZ/MCl_x_ was studied by DSC and the curves are shown in Figure 2 and Table 2. As shown in Figure 2A, the exothermic peak temperature (T_p_) of BZ is 192.8°C. For PBZ-M, broad exothermic peaks are observed. The exothermic peak temperatures decreased from 192.8 to 134.2°C, 124.5, and 109.4°C for BZ/CuCl_2_, BZ/FeCl_3,_ and BZ/AlCl_3_, respectively. The results indicate that MCl_x_ can effectively catalyze the polymerization of BZ. Furthermore, no isothermal DSC curves were used to study the curing kinetics of BZ and BZ/MCl_x_ at different rates (*β* = 5, 10, 15°C min^−1^). The results are shown in Figures 2C,D and Supplementary Figures S3,S4. The activation energy (E_a_) is calculated using Kissinger (Kissinger 1957) and Ozawa (Takeo 1965) models. The activation energy of BZ/MCl_x_ is dramatically decreased compared with BZ. This means that BZ/MCl_x_ is easier to activate and polymerize than BZ.

**TABLE 2:** Curing parameters of benzoxazine monomers from non-isothermal DSC experiments.

Sample	T_onset_ (°C)	T_p_ (°C)	E_a1_/kJ·mol^−1^	E_a2_/kJ·mol^−1^
PBZ	150	193	87.6	90.6
PBZ-Cu	85	134	43.8	50.5
PBZ-Al	70	110	41.9	48.2
PBZ-Fe	81	124	42.9	49.4

E_a1_: Kissinger method.

E_a2_: Ozawa method.

### Characterization of Polybenzoxazine

**FIGURE 3:**
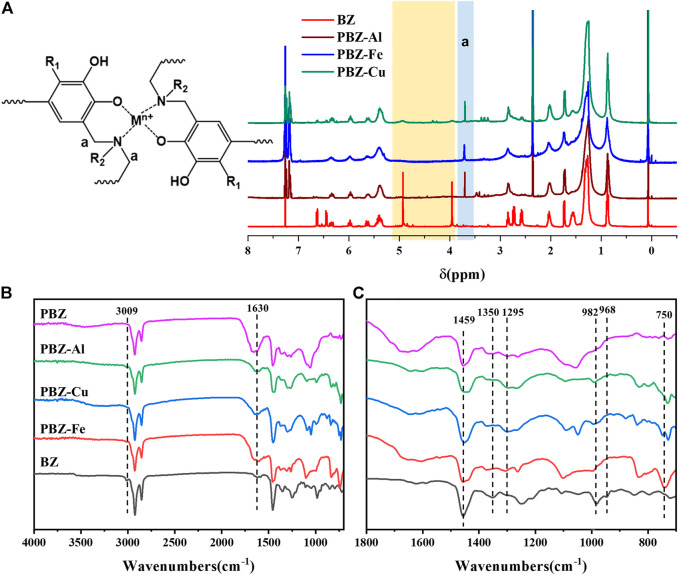
NMR **(A)** and ATR-FTIR **(B,C)** spectrum of BZ and PBZ-M.

Metal ionic catalytic polymerization of PBZ-M was examined by NMR and ATR-FTIR. Figure 3A and Supplementary Figure S5 show the comparison of NMR spectra of PBZ-M and BZ. Compared to BZ, the peaks assigned to protons in Ar–CH_2_–N and O–CH_2_–N in PBZ-M disappeared and a new peak assigned to the Mannich bridge appeared. Figures 3B,C show the ATR-FTIR spectra of PBZ-M, PBZ, and BZ. The band at 968 cm^−1^, characteristic absorption peaks of benzoxazine oxazine ring, dramatically decreases. In addition, the stretching vibration absorption peak of Ar-O shifts from 1,350 cm^−1^ to 1,295 cm^−1^ because of that the structure of Ar-O-C is converted into the structure of Ar-O-H during the ring-opening reaction of the oxazine. The abovementioned changes indicate that the MCl_x_ can promote benzoxazine’s ring-opening reaction. The band at 3,009 cm^−1^ assigned to the stretching vibration of the isolated double bond is weakened, indicating that the isolated double bond in the alkyl side group is also involved in the polymerization reaction. The band at 3,200–3,600 cm^−1^ assigned to free (–OH) in the monomer is weak. After polymerization, the shoulder at 3,200–3,600 cm^−1^ is still weak in PBZ-Cu and PBZ-Fe. In contrast, a broad band at 3,200–3,600 cm^−1^ appeared in PBZ-Al. The possible explanation for this unexpected difference between PBZ-Al and PBZ-Cu and PBZ-Fe is that Cu^2+^ and Fe^3+^ are oxidizing agents. The phenolic hydroxyl groups generated during polymerization were oxidized to carbonyl groups. It can also be further demonstrated by the stronger intensity of the peak at 1,630 cm^−1^ that is assigned to the carbonyl groups in PBZ-M than that of PBZ. Another reason for this difference may be that the coordination situation of the metal and the hydroxyl group in PBZ-M is different. We boldly assume that the Al^3+^ in PBZ-Al is not mainly coordinated with hydroxyl groups while the Cu^2+^ and Fe^3+^ is.

### XPS

**FIGURE 4:**
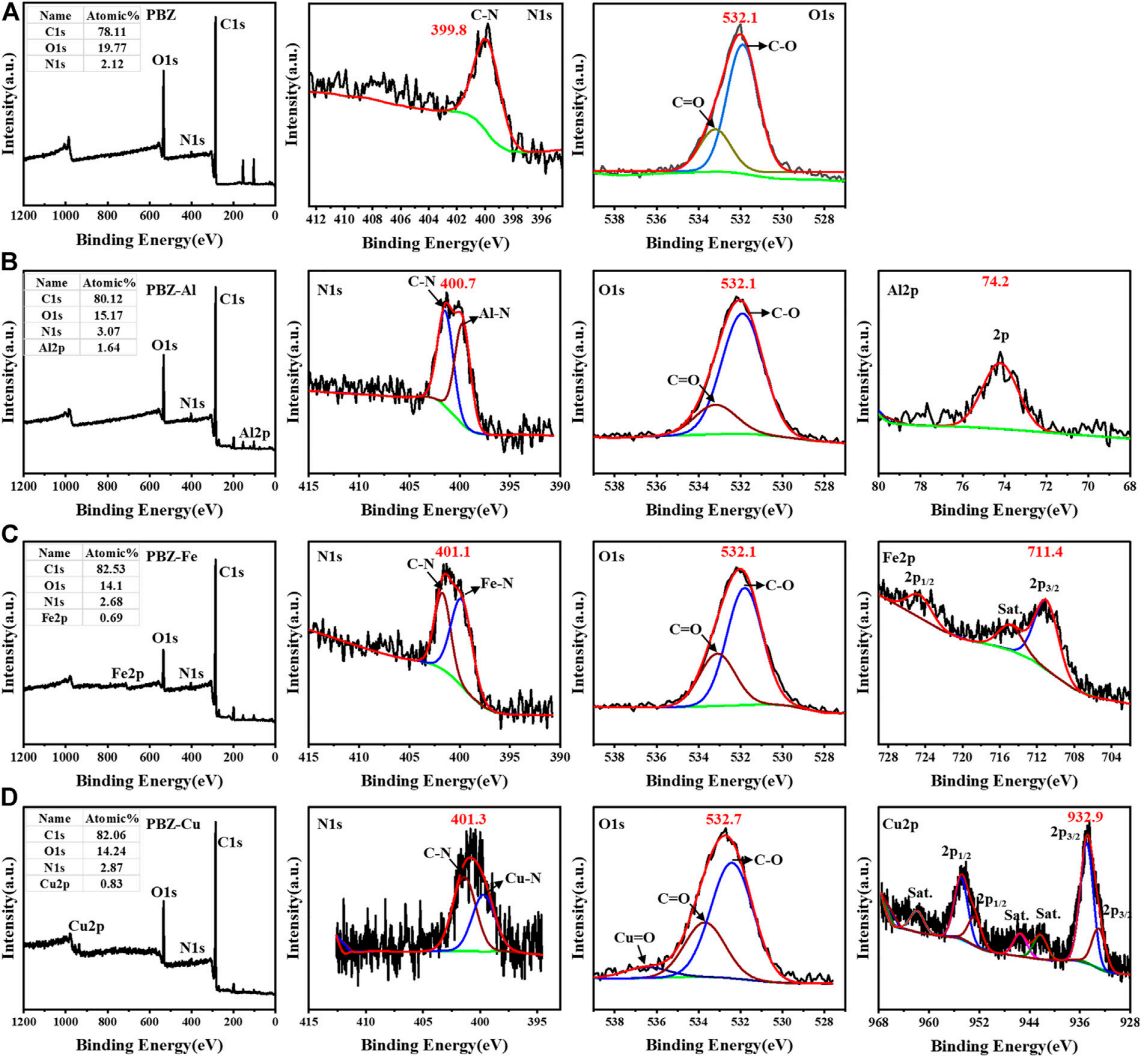
Xps of the PBZ **(A)** and PBZ-M **(B–D)**.

To reveal the interaction of the metal ion and the polymers, XPS was conducted to show the element distribution in the polymer. Figure 4 shows the XPS spectra of PBZ, PBZ-Al, PBZ-Cu, and PBZ-Fe. In the XPS spectra of PBZ, there are only three peeks relating to O1s (532.1 eV), N1s (399.8 eV), and C1s (284.6 eV). In the XPS spectra of PBZ-Al (Kurdi et al., 2002), there are four peeks relating to O1s (532.1 eV), N1s (400.7 eV), C1s (284.6 eV), and Al2p (74.2 eV). In the XPS spectra of PBZ-Cu(Dong et al., 2011), there are four peeks relating to O1s (532.7 eV), N1s (403.1 eV), C1s (284.6 eV), and Cu2p (932.9 eV). In the XPS spectra of PBZ-Fe (Deng et al., 2017), there are four peeks relating to O1s (532.1 eV), N1s (401.1 eV), C1s (284.6 eV), and Fe2p (711.4 eV). The feature peak position and the change of the polymer electron binding energy of elements are summarized in Table 3. Compared to the binding energy of the metal elements in MCl_x_, the binding energy of the metal elements in PBZ-M are all changed (△_Al_ = −0.6 eV, △_Al_ = −0.2 eV, △_Cu_ = −1.3 eV). It indicates the interaction between metal ions and the main structure of benzoxazine after the polymerization. In addition, the change of the binding energy of Cu^2+^ is larger than Fe^3+^ and then Al^3+^, indicating a stronger interaction between Cu^2+^ and benzoxazine. The binding energy of N is also changed (△_N-PBZ-Al_ = 1.3 eV, △_N-PBZ-Cu_ = 1.5 eV, and △_N-PBZ-Al_ = 0.9 eV). As for the element of oxygen, the binding energy of PBZ-Al and PBZ-Fe is not changed compared with that of PBZ. The binding energy of PBZ-Cu increased from 532.1 to 532.7 eV (△_O-PBZ-Cu_ = 0.6 eV). The abovementioned analysis indicates Cu^2+^ is coordinated with both oxygen and nitrogen atoms while the Fe^3+^ and Al^3+^ are coordinated with only nitrogen atoms.

**TABLE 3:** The XPS analysis of PBZ and PBZ-M.

Polymer	Peak BE					MCl_x_	M_2p_	N_1s_	O_1s_
	C_1s_	O_1s_	N_1s_	Cl_2p_	M_2p_/eV	M_2p_/eV	△/eV	△/eV	△/eV
PBZ	284.6	532.1	399.8	—	—	—	—	—	—
PBZ-Fe	284.7	532.1	401.1	199.5	711.4	711.99	−0.6	1.3	0
PBZ-Al	284.6	532.1	400.7	199.5	74.2	74.4	−0.2	0.9	0
PBZ-Cu	284.6	532.7	401.3	199.4	932.9	934.2	−1.3	1.5	0.6

### Mechanical Performance

The effect of metal ions on the mechanical properties of PBZ-M is shown in Table 4. Overall, PBZ-Cu has better mechanical property (hardness = 2H, impact resistance = 40 cm, flexibility = 1 mm and adhesion = grade 2) than PBZ-Fe and PBZ-Al. The impact resistance and flexibility of PBZ-Fe and PBZ-Al are similar. PBZ-Fe is harder than PBZ-Al, while the adhesion is poorer than PBZ-Al.

**TABLE 4:** The comparison of the mechanical properties of PBZ-Cu, PBZ-Al, PBZ-Fe, and PBZ.

Properties	PBZ-Fe	PBZ-Al	PBZ-Cu	PBZ
Reaction time (h)	6	6	6	—
Surface drying time (min)^a^	30	30	30	—
Full dry (h)^b^	<2	<2	<2	—
Hardness	H	HB	2H	6H
Impact resistance (cm)	20	20	40	15
Flexibility (mm)	10	10	5	>10
Adhesion (grade)	5	3	3	2

a The surface drying state of a coating when Ballotini (small glass spheres) can be lightly brushed away without damaging the surface of the coating (ISO 1517-1973).

b The condition of the film in which it is dry throughout its thickness (ISO 9117-1990).

### Surface’s Wettability

**FIGURE 5:**
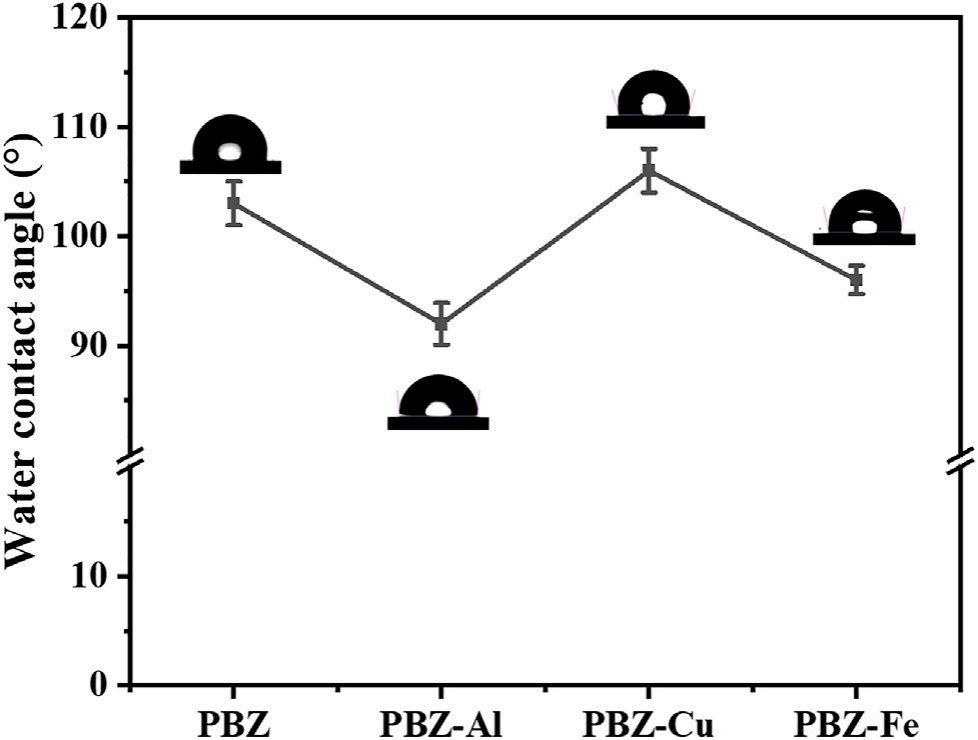
Water contact angles of the PBZ and PBZ-M.

Due to the strong intramolecular hydrogen bonding between the hydroxy groups of polybenzoxazines, its surface energy is even lower than pure polytetrafluoroethylene (PTFE). To reveal the influence of the metal ion on the wettability of the polymer, we measured the water contact angle (WCAs) of PBZ, PBZ-Cu, PBZ-Fe, and PBZ-Al. As shown in Figure 5, compared with PBZ (WCA_PBZ_ = 103° ± 2°), the introduction of Al^3+^ and Fe^3+^ reduces the WCA (WCA_PBZ-Al_ = 92° ± 1.9° and WCA_PBZ-Fe_ = 96° ± 1.3°). The reason for the negative effect of Al^3+^ and Fe^3+^ on the WCAs of PBZ-Al and PBZ-Fe is the chelation between Al^3+^ and Fe^3+^ and the nitrogen atom, destroying the intramolecular hydrogen bonding of the hydroxy groups. In contrast, the intramolecular hydrogen bonding of the hydroxy groups is replaced by the chelation between Cu^2+^ and the nitrogen atom and oxygen atom. The introduction of Cu^2+^ has a positive effect on the WCA (WCA_PBZ-Cu_ = 106° ± 2°).

### Thermal Stability

**FIGURE 6:**
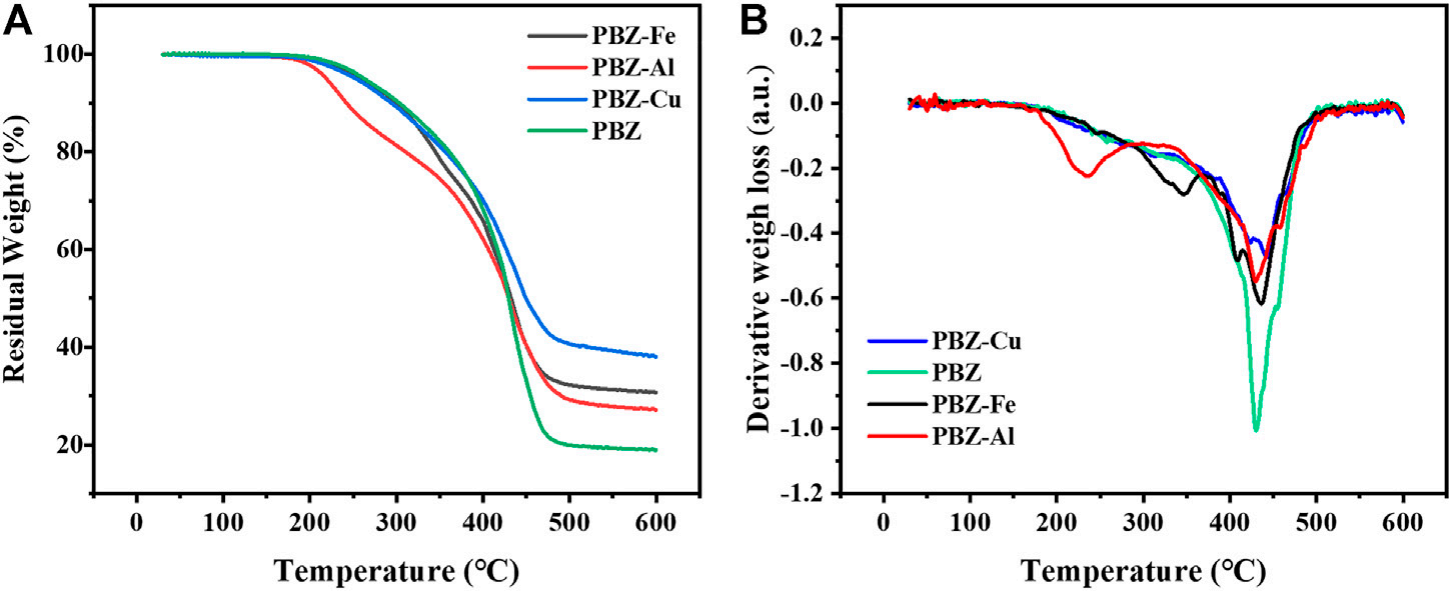
TGA traces **(A)** and DTA curve **(B)** of the PBZ-M.

The effect of the metal ions catalyst on the thermal stability was studied by using thermogravimetric analysis (TGA). Figure 6 shows the TGA traces and DTG curves of PBZ, PBZ-Fe, PBZ-Al, and PBZ-Cu. The related thermogravimetric results are presented in Table 5. As we can see, the onset degradation temperature of these polymers is almost the same (∼200°C). The char yield of PBZ at 600°C is 19.20%. The char yield of PBZ-M is effectively improved compared to PBZ. The char yields of cured PBZ-Al, PBZ-Fe, and PBZ-Cu are increased from 19.20 to 27.42, 31.23, and 38.34%, respectively. Accordingly, PBZ-Cu exhibits better thermal stability than PBZ-Fe and PBZ-Al as reflected by *T*_*10%*_, *T*_*50%*_, and *T*_max_ values. The effect of metal ions on improving the thermal stability of benzoxazine can be explained as follows: 1) the mechanical properties (hardness, listed in Table 3) and the gel fraction of PBZ-Al (HB, 84.3), PBZ-Fe (H, 85.3) and PBZ-Cu (2H, 87.4) are increased, indicating that the degree of crosslinking is increased. The higher degree of crosslinking, the more stable the PBZ-M is. 2) Cu^2+^ and Fe^3+^ are variable valence metal ions while Al^3+^ is not, indicating that Cu^2+^ and Fe^3+^ can oxidize the polybenzoxazine. In the oxidation of polybenzoxazine, the Mannich base transforms into an amide and/or imide-like structure, which is a thermally more stable structure. 3) According to the results of XPS analysis, PBZ-Cu has chelation between Cu^2+^ and nitrogen (N) and oxygen (O) atoms. PBZ-Fe and PBZ-Al have chelation between M^3+^ and oxygen (O) atom. The coordination bond strength in PBZ-Cu is higher than that in PBZ-Fe and PBZ-Al. It can be concluded from the abovementioned results that the thermal stability of polybenzoxazines treated with metals can be affected by the chemical structures of the polymers.

**TABLE 5:** Thermal properties of PBZ, PBZ-Cu, PBZ-Al, and PBZ-Fe.

Sample	T_10%_ (°C)	T_50%_ (°C)	T_f_/°c	T_max_ (°C)
PBZ	300	430	—	432
PBZ-Cu	300	450	—	440
PBZ-Al	240	430	240°C	430
PBZ-Fe	300	430	360°C	435

T_10%_: temperature corresponding to 10% mass losses.

T_50%_: temperature corresponding to 50% mass losses.

T_f_: the first peak temperature of degradation for organic ligand in polymers.

T_max_: temperature for maximum weight loss extracted from DTA, graph.

## Conclusion

The urushiol-based polybenzoxazine was prepared with natural urushiol, formaldehyde, and *n*-octylamine and then cured with the Lewis acid (FeCl_3_, AlCl_3_, and CuCl_2_) at low temperature. The polymer film can be prepared at room temperature instead of high-temperature treatment. The effect of the Lewis acid on structures and properties of the polymers is revealed. The results indicate that Cu^2+^ is coordinated with both oxygen and nitrogen atoms while the Fe^3+^ and Al^3+^ are coordinated with only nitrogen atoms. The effect of the Lewis acid on the mechanical properties, wettability, and thermal stability was investigated. PBZ-Cu has better mechanical properties than PBZ-Fe and PBZ-Al. The introduction of Cu^2+^ has a positive effect on the WCA while the introduction of Fe^3+^ and Al^3+^ has a negative effect. The thermal stability is significantly improved from 19.2% (PBZ) to 38.34% (PBZ-Cu). We hope that this study will expand the application of polybenzoxazine.

## Data Availability

The original contributions presented in the study are included in the article/Supplementary Material, further inquiries can be directed to the corresponding authors.
