# Supplementary material for: Metal Ion-Catalyzed Low-Temperature Curing of Urushiol-Based Polybenzoxazine
Source: Front Chem. 2022 Apr 28;10:879605. doi: 10.3389/fchem.2022.879605 (PMC9096162; doi:10.3389/fchem.2022.879605)
Supplement: Supplementary file 1 [file DataSheet1.docx]

Supplementary Material

Metal ion-catalyzed low-temperature curing of urushiol-based polybenzoxazine

Wen Yang^1^, Yaofeng Xie^1^, Jipeng Chen^1*^, Chunmei Huang^1^, Yanlian Xu^2*^, Yucai Lin^1,3,4*^

^1^College of Chemistry and Materials, Fujian Normal University, Fuzhou, 350007, PR China

^2^Fujian Engineering Research Center of New Chinese Lacquer Materials, Minjiang University, Fuzhou, 350108, PR China

^3^Fujian Key Laboratory of Polymer Materials, Fujian Normal University, Fuzhou, 350007, PR China

^4^Fujian Provincial Key Laboratory of Advanced Oriented Chemical Engineering, Fujian Normal University, Fuzhou, 350007, PR China

*** Correspondence:**Yucai Lin
yucailin@fjnu.edu.cn

Yanlian Xu
ylxu@mju.edu.cn

Jipeng Chen


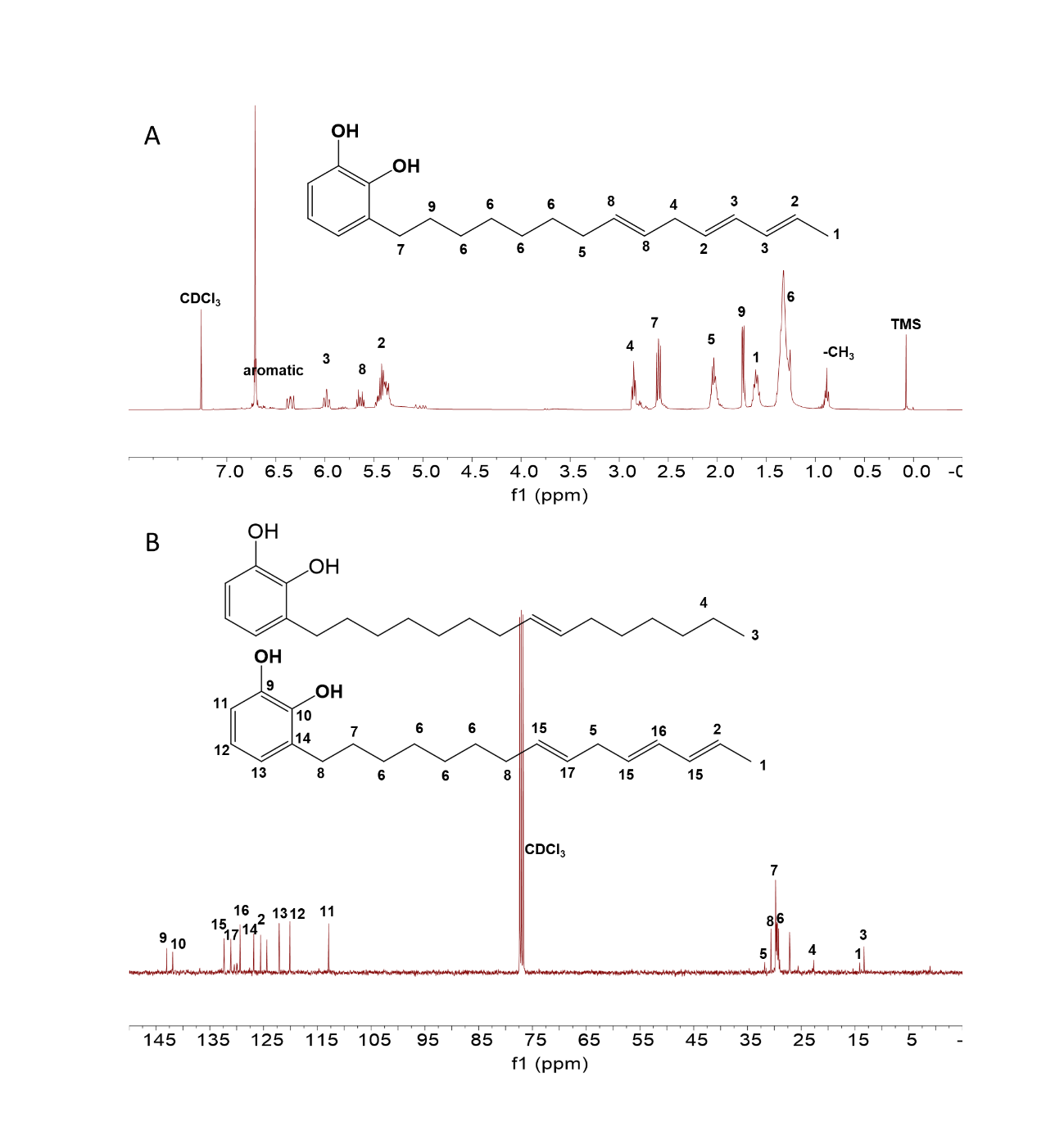


**Figure S1.** NMR spectrum of urushiol.


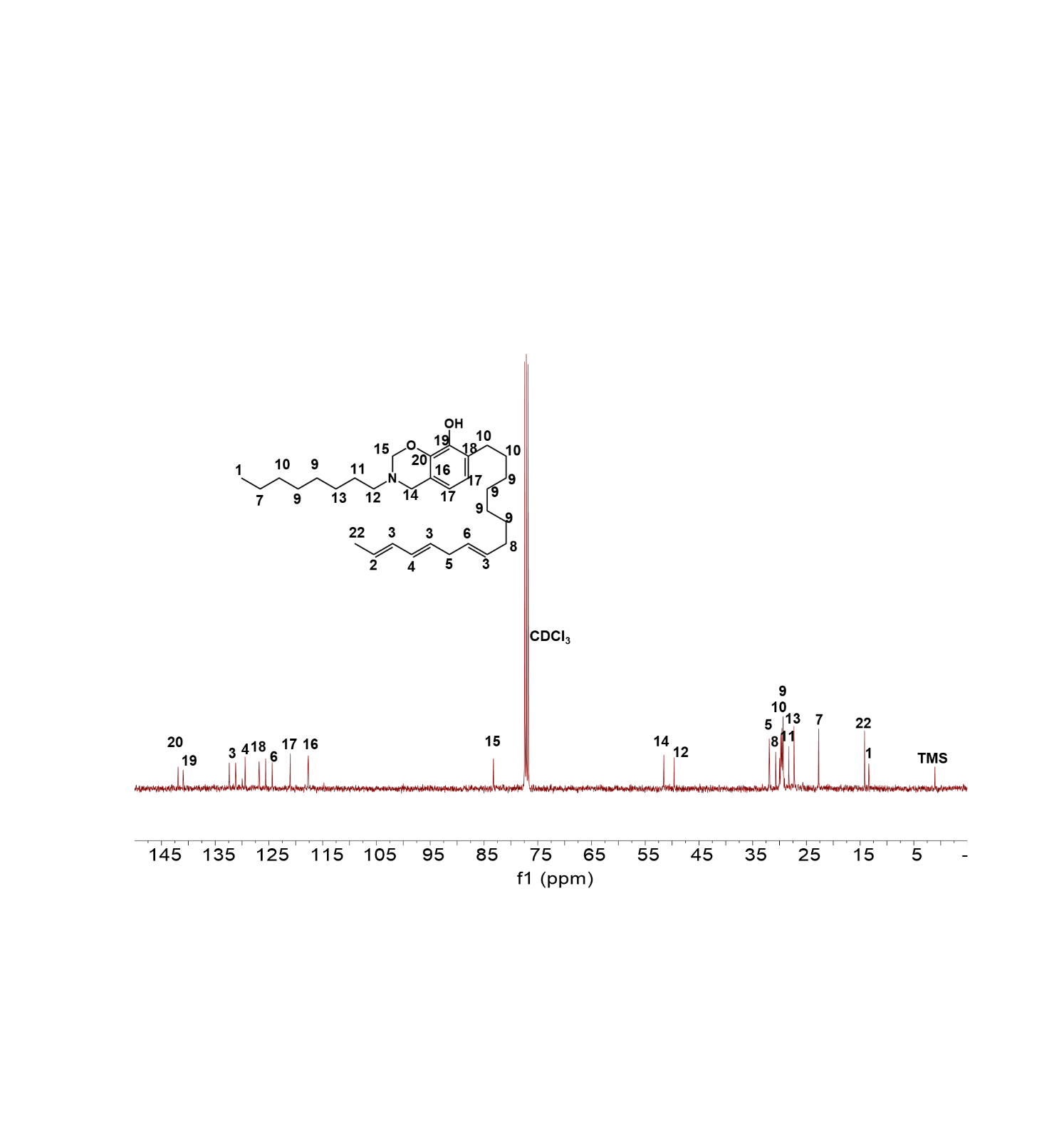


**Figure S2.** ^13^C-NMR spectrum of BZ.


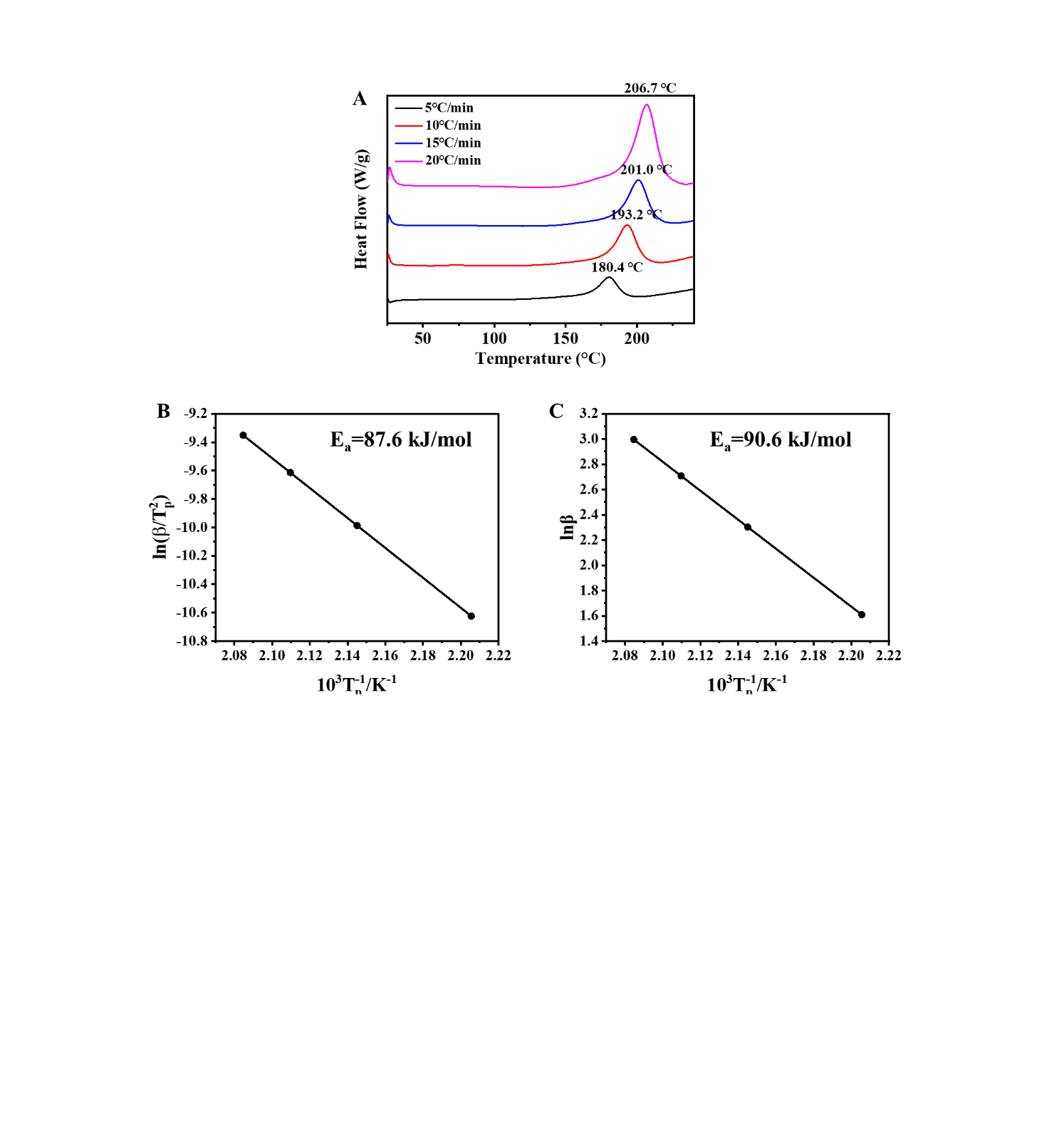


**Figure S3.** (A) The curing kinetics of BZ at different rates (β=5, 10, 15, 20 °C min^−1^). (B,C) The activation energy (E_a_) calculated using Kissinger (B) and Ozawa (C) models.


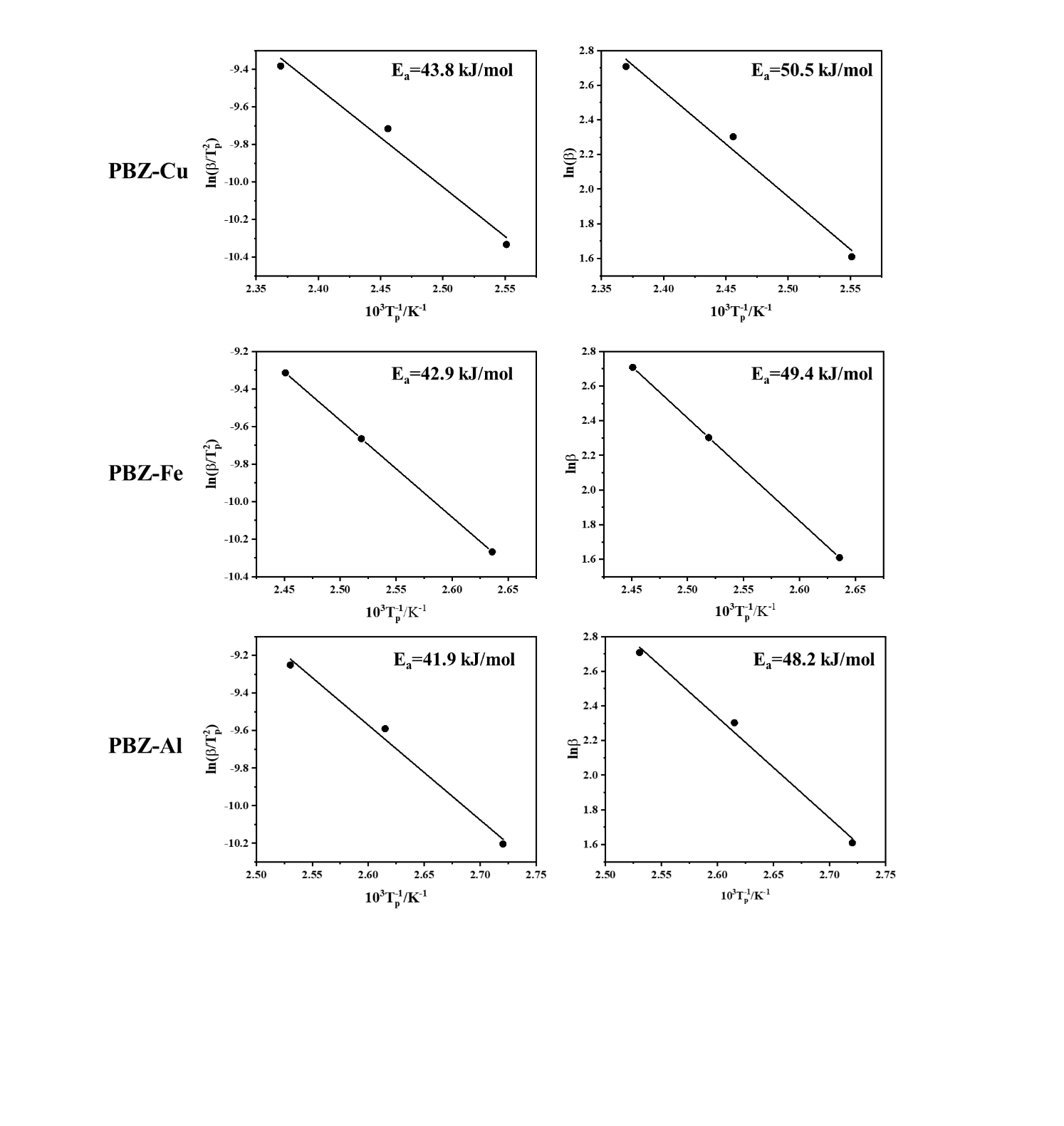


**Figure S4.** The activation energy (E_a_) of PBZ-Cu, PBZ-Fe and PBZ-Al calculated using Kissinger and Ozawa models.


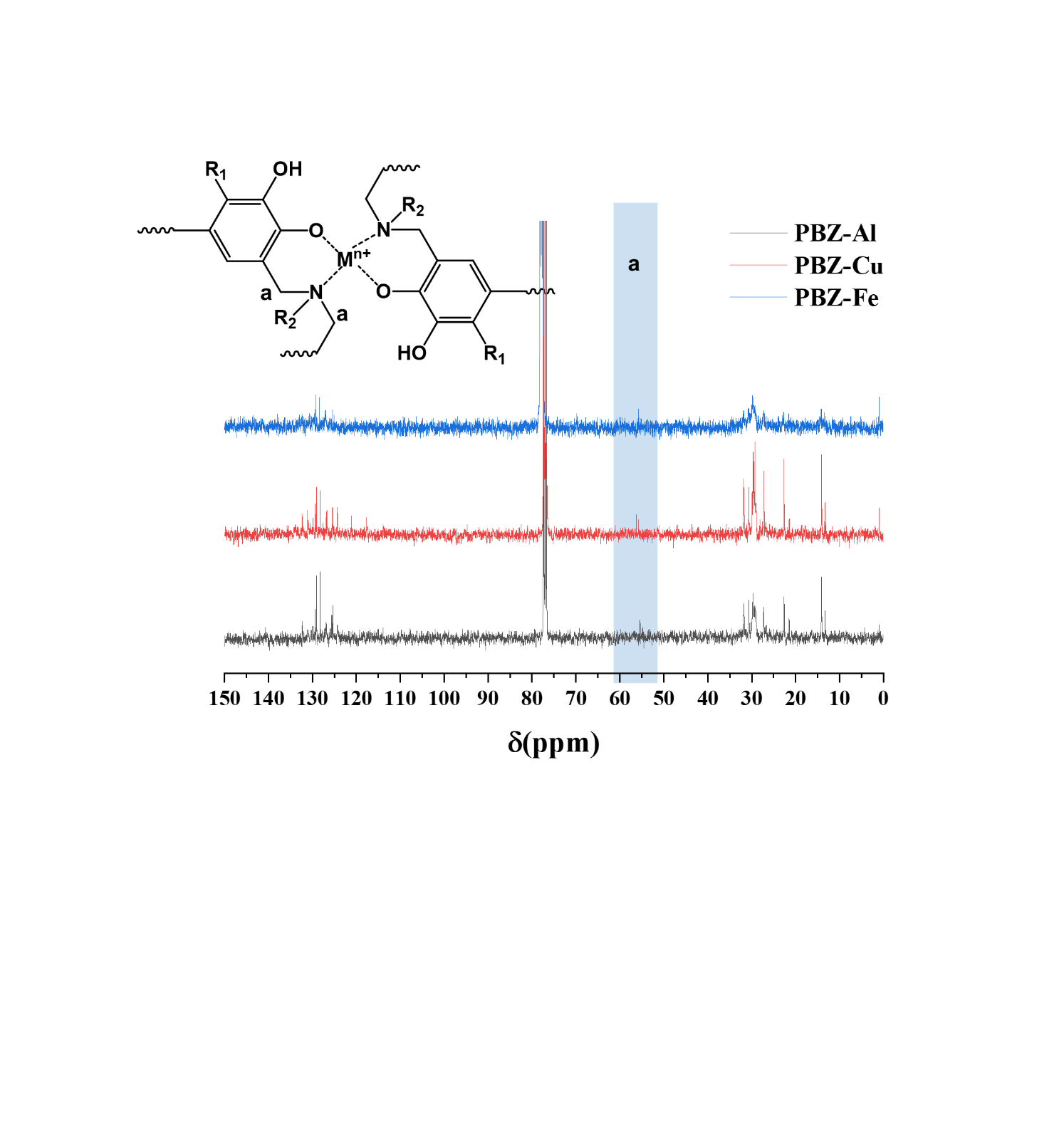


**Figure S5.** ^13^C-NMR spectrum of PBZ-Al, PBZ-Cu and PBZ-Fe.
